# Application of machine learning techniques to automate speech mispronunciation detection in children and adolescents

**DOI:** 10.1590/1678-7765-2026-0078

**Published:** 2026-09-07

**Authors:** Nazila Ameli, Mahshid Nik Ravesh, Luan Matheus Trindade Dalmazo, Karen Pollock, Daniel DeSantis, Manuel Lagravere, Hollis Lai

**Affiliations:** 1 University of Alberta Mike Petryk School of Dentistry Edmonton Alberta Canada University of Alberta, Mike Petryk School of Dentistry, Edmonton, Alberta, Canada; 2 Autonomous Researcher Toronto Ontario Canada Autonomous Researcher, Toronto, Ontario, Canada; 3 Autonomous Researcher Edmonton Alberta Canada Autonomous Researcher, Edmonton, Alberta, Canada

**Keywords:** Speech sound errors, Mispronunciation errors, Machine learning, Deep learning, Pediatrics

## Abstract

**Introduction:**

Speech sound disorders (SSDs) are common in children and can affect academic and psychosocial outcomes. Clinical identification relies on expert auditory-perceptual assessment, which is time-intensive and may vary across raters. Automated screening tools could support triage when access to specialists is limited.

**Objective:**

This study aimed to develop and evaluate a deep learning (DL) classifier for detecting mispronunciation errors in standardized pediatric speech recordings obtained using a structured fricative-focused word elicitation protocol, using expert-adjudicated labels as the reference standard.

**Methodology:**

In this cross-sectional study, we analyzed 100 participants (6–18 years) providing 1,800 standardized word recordings. Two expert speech-language pathologists (SLPs) labeled recordings as mispronunciation present vs. absent; disagreements were adjudicated by a third SLP. Inter- and intra-rater reliability were assessed. Audio was denoised and standardized to 16 kHz. A pretrained transformer speech model (WavLM Base+) was fine-tuned for recording-level binary classification. Class imbalance was addressed using augmentation, weighted sampling, and focal loss. Performance was assessed on a held-out test set at the recording level using accuracy, sensitivity, specificity, precision, F1-score, and area under the ROC curve (AUC).

**Results:**

The overall recording-level prevalence of speech sound errors was 16.15%, with the “th” category (/θ/ + /ð/) being the most frequently mispronounced phoneme group. No significant differences were observed by sex, age group (6–9, 10–13, 14–18 years), or malocclusion status (p > 0.05). Labeling showed strong agreement (Cohen’s κ = 0.84; intra-rater reliability 0.89–0.92). On the test set (253 recordings), the model achieved 90.9% accuracy, 77.8% sensitivity, 98.2% specificity, 95.9% precision, and AUC = 0.936.

**Conclusions:**

Fine-tuned pretrained speech representations demonstrated promising performance for screening pediatric mispronunciation errors under standardized recording conditions using expert labels. The high-specificity profile supports its use as a screening and triage decision-support tool, while future studies are needed to validate performance across broader clinical and real-world settings.

## Graphical abstract

**Figure:**
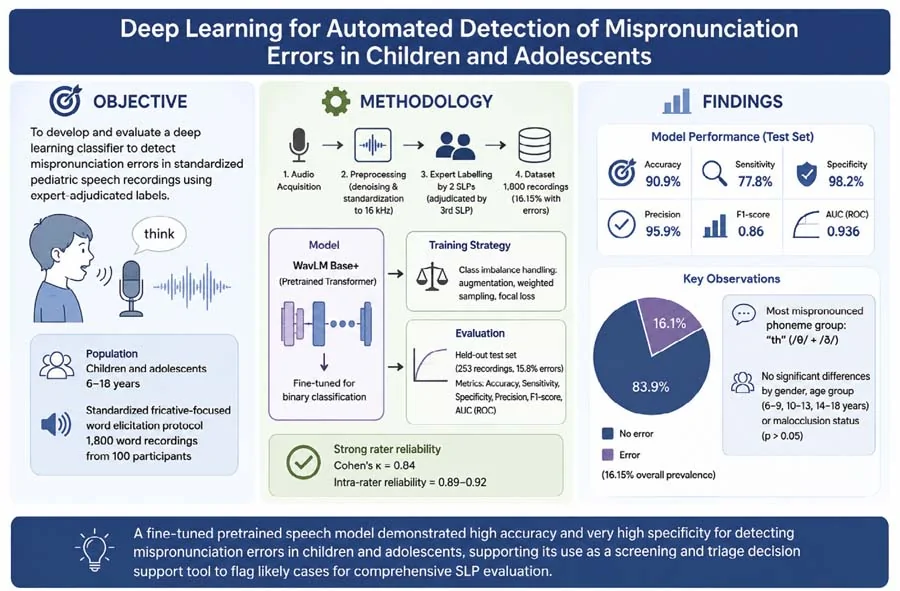

## Introduction

Speech sound disorders (SSDs) are among the most common communication impairments in children, affecting approximately 48.1% of school-aged children.^1^ SSDs encompass difficulties in the perception, motor production, or phonological representation of speech sounds, and they may persist into adolescence when not identified and managed early.^2^ Reduced speech intelligibility can negatively impact academic performance, literacy development, and classroom participation, and may also affect self-esteem and social integration.^3,4^ These challenges can extend into adulthood, with potential long-term effects on employment opportunities and daily communication.^5^

SSDs arise from diverse etiologies, including structural craniofacial conditions (e.g., cleft palate), neurological disorders (e.g., cerebral palsy), functional impairments affecting oral-motor coordination, environmental or behavioral factors, and malocclusions.^6-16^ Importantly, speech sound errors and mispronunciation patterns are multifactorial phenomena and are not specific to orthodontic or malocclusion-related conditions, as many children with SSDs present without dentofacial abnormalities, while many individuals with malocclusion demonstrate typical speech production.^1,6,12^ In current practice, SSD diagnosis primarily relies on auditory-perceptual evaluation by speech-language pathologists (SLPs), often supplemented by standardized word lists and phonetic transcription.^17^ While clinically essential, these assessments are time-intensive and can be influenced by rater variability, fatigue, and environmental factors, potentially introducing inconsistency across evaluations.^17-19^ Access barriers may further delay timely assessment, particularly in settings with limited SLP availability. In addition, speech and dental assessments are frequently conducted within separate clinical workflows, which can complicate interdisciplinary coordination and delay early referral when both orthodontic and speech-language interventions may be indicated.^20^

Artificial intelligence (AI) and machine learning (ML) offer an opportunity to support more scalable, objective screening in domains driven by pattern recognition.^21^ Deep learning (DL) approaches have shown strong performance in speech-related tasks, including speech recognition and speech error detection, by learning complex acoustic representations from audio data.^22,23^ However, DL applications focused specifically on identifying mispronunciation errors in children, particularly in a way that could support dental and interdisciplinary contexts, remain comparatively underexplored, and most existing systems emphasize neurological or general speech disorders rather than articulation-level mispronunciation patterns relevant to clinical screening.^6,24^ Because phoneme production and articulation norms are language-dependent, the present study specifically focused on standardized English-language speech stimuli and the findings are therefore not intended to imply universal phonological applicability across other languages.

Thus, the objective of this study was to develop and evaluate a DL model for the detection of mispronunciation errors in standardized pediatric speech recordings obtained using a structured fricative-focused lexical and phonemic elicitation protocol, using expert SLP-adjudicated labels as the reference standard. This study was designed as a screening-support investigation focused on articulation-level mispronunciation detection rather than comprehensive SSD diagnosis. In addition, this work is positioned for potential interdisciplinary screening-support applications in dental and orthodontic settings, where children are routinely assessed and where early identification of possible speech concerns may support referral for comprehensive speech-language evaluation. By providing an objective and efficient screening approach, this work aims to complement, rather than replace, expert clinical evaluation. Because children frequently present to dental and orthodontic clinics before receiving speech-language evaluation, such screening tools may help identify unmet speech needs within routine dental workflows. Such a tool may support triage for comprehensive SLP assessment, enable earlier identification in settings with limited access to specialists, and facilitate interdisciplinary communication when speech concerns arise in dental care contexts.

## Methodology

### Study design and participants

This cross-sectional study was approved by the Health Ethics Research Board (Pro00133197). Written and verbal informed consent was also obtained from participants and their parents/guardians prior to participation. Assent was also obtained from participating children and adolescents according to their age and level of understanding. All participants and their families were informed about the study procedures before enrollment, and participation was voluntary. All data were collected, stored, and processed in compliance with institutional privacy and confidentiality regulations. This study developed and evaluated a DL classifier to detect mispronunciation errors in short speech recordings. The study population included 100 children and adolescents (53 female, 47 male) aged 6–18 years (mean 10.37 years, SD 2.51). Participants were recruited through voluntary convenience sampling from pediatric dental and orthodontic clinics, where children and adolescents attending routine dental or orthodontic consultations were invited to participate in the study together with their parents/guardians. To better characterize the age distribution, participants were grouped into three developmental categories: 6–9 years (n = 35), 10–13 years (n = 48), and 14–18 years (n = 17), reflecting early childhood, middle childhood, and adolescence, respectively, to account for potential developmental differences in speech production. Participants were not required to be native English speakers. Bilingual and multilingual participants were eligible for inclusion provided they demonstrated functional English proficiency sufficient to complete the standardized English speech tasks. All participants had resided in Canada for at least four years prior to enrollment and regularly used English in educational and social environments. No formal exclusion criteria related to bilingualism or multilingualism exposure were applied. All 100 recruited participants underwent a standardized orthodontic clinical examination. The primary dental and occlusal findings were initially assessed by trained dental students using predefined criteria and subsequently confirmed by an experienced orthodontist with more than five years of clinical orthodontic practice. Occlusal assessment was based on clinical examination and supported by panoramic radiographs. Occlusal characteristics were categorized as normal occlusion, increased overjet (more than 4 mm), crowding (moderate to severe: greater than 4 mm),^25^ anterior open bite, posterior or anterior crossbite, or deep overbite. For subgroup analyses, participants were categorized as “malocclusion present” if any occlusal finding other than normal occlusion was identified; otherwise, they were categorized as “no malocclusion.”

Then, speech samples were recorded in a sound-treated room using a standardized word list targeting fricative consonants. Target words were presented using a standardized slide presentation that included both a visual representation (image of the object) and the written form of each word. This dual-modality presentation ensured that participants of all ages could reliably identify and produce the target words without requiring reading proficiency, and no participant required the clinician to repeat any word. Each word was elicited once under these standardized conditions.

Recordings were obtained with participants seated at a fixed distance from the recording device (approximately one chair’s distance from the computer). Audio was captured using the built-in microphone of the recording device without the use of an external microphone, and identical recording conditions were maintained across all participants.

Six English fricative consonant categories (/s/, “th”, /z/, /v/, /f/, and /ʃ/) were selected because fricatives require precise articulatory positioning and airflow control, making them clinically informative for evaluating articulation distortions within standardized speech elicitation tasks. The selected categories were chosen to provide representation across common English fricative place-of-articulation patterns, including labiodental (/f/, /v/), alveolar (/s/, /z/), dental (“th”: /θ/ and /ð/), and postalveolar (/ʃ/) fricatives. Importantly, the rationale for inclusion was based primarily on articulatory configuration and airflow characteristics rather than voicing distinctions alone, since voiced-voiceless fricative pairs generally share similar articulatory placement.

In the present study, the “th” category was operationally treated as a grouped dental fricative category including both the voiceless dental fricative /θ/ (e.g., “thumb” and “bath”) and the voiced dental fricative /ð/ (e.g., “feather”). Similarly, the “sh” category corresponded to the voiceless postalveolar fricative /ʃ/. The voiced postalveolar fricative /ʒ/ shares the same place of articulation (postalveolar) and manner of articulation (fricative) as /ʃ/, differing only in voicing; however, /ʒ/ was not included as a target phoneme in the present elicitation protocol. The protocol was intentionally designed as a focused fricative-based screening-oriented elicitation task rather than a comprehensive assessment of the full English phonological inventory. Selection of target phonemes was additionally informed by established pediatric articulation assessment frameworks, which commonly evaluate speech production across multiple word positions using clinically informative consonant categories relevant to airflow precision and articulatory placement.^26,27^

For each phoneme category, three standardized English-language target words were included, with the fricative occurring in initial, medial, and final phoneme positions, for a total of 18 words per participant (Appendix 1), yielding 1,800 audio recordings. This structured elicitation approach was consistent with established pediatric speech assessment frameworks that evaluate phoneme production across word positions to capture context-dependent variability in articulation.^26,27^

Participants with a known history of diagnosed SSD, significant articulatory or phonological difficulties, current or previous speech-language therapy, congenital craniofacial anomalies, or hearing impairments were excluded. The purpose of these exclusions was to avoid overrepresentation of previously diagnosed or clinically severe speech disorders and instead evaluate whether the proposed model could detect naturally occurring mispronunciation errors and subtle articulation deviations within a general pediatric population under standardized screening conditions. Consequently, the study was designed as a screening-oriented proof-of-concept investigation rather than a diagnostic study of clinically confirmed SSD populations.

### Speech labeling and reliability assessment

Each recording was independently labeled by two expert SLPs as mispronunciation error present or absent. A mispronunciation error was defined as any perceptually identified deviation from the expected target phoneme production based on auditory-perceptual evaluation using standard clinical criteria. This included distortions, substitutions, omissions, and atypical articulatory productions identified by expert SLP raters. Because the objective of the study was to develop a binary screening-oriented classifier (mispronunciation error present vs. absent), individual error subtypes were not separately coded or analyzed quantitatively. When the two raters disagreed, the recording was reviewed by a third expert SLP, and the adjudicated label was used as the final reference standard.

Both inter-rater and intra-rater reliability were assessed. For intra-rater reliability, the primary SLP raters repeated the labeling of 20 randomly selected recordings after a two-week washout period using the same standardized criteria.

### Audio denoising and preprocessing

All audio recordings were preprocessed prior to feature extraction. Waveform-level noise reduction was applied using the noisereduce Python library, which implements spectral gating-based noise suppression. Each audio file was loaded using librosa (preserving the original sampling rate), and background noise was reduced using the reduce_noise function with default parameters, applied uniformly across all recordings. The denoised waveforms were then saved and used for subsequent model input. This preprocessing step was intended to reduce background noise while preserving speech-relevant acoustic features.

Identical preprocessing was applied to all recordings to ensure consistency across participants and classes. For model input, audio was standardized to a 16 kHz sampling rate. Recordings of variable duration were handled using dynamic padding during feature extraction to enable minibatch training.

### Dataset composition, augmentation, and data splits

The primary (non-augmented) dataset comprised 1,345 recordings labeled as without mispronunciation error and 455 labeled with mispronunciation error. Each participant produced 18-word recordings, comprising three target words for each fricative category: labiodental (/f/, /v/), alveolar (/s/, /z/), dental (“th”: /θ/ and /ð/), and postalveolar (/ʃ/) fricatives. Because speech sound errors were phoneme-specific and could vary across individual word productions within the same participant, the unit of analysis for model training and evaluation was the individual recording rather than the participant. Accordingly, the dataset split was performed at the recording level, meaning that individual audio recordings, rather than entire participants, were randomly assigned to training (70%), validation (15%), and test (15%) subsets. As a result, recordings originating from the same participant could be present across different dataset partitions.

Data augmentation was applied only within the training subset and included time shift, additive noise, gain adjustment, and time-stretch/pitch transformations, resulting in final sample counts of 2,608 (training), 265 (validation), and 253 (test).

### Model development and training

A transfer learning approach was used by fine-tuning WavLM Base+ (microsoft/wavlm-base-plus), a transformer-based pretrained speech representation model, for binary audio classification.^28,29^ Denoised waveforms were processed using the corresponding Hugging Face feature extractor with padding enabled. The model consisted of a pretrained WavLM encoder with a sequence-classification head producing classification logits for two classes (mispronunciation error present vs. absent), and all parameters were fine-tuned end-to-end.^30^

Training was performed using AdamW optimization (learning rate = 1×10^-^5, batch size = 4) for up to 20 epochs. Early stopping with a patience of two epochs was applied based on validation accuracy, and the model achieving the highest validation performance was saved and used for testing. No learning-rate scheduler was employed, and no explicit weight decay value was specified (default AdamW settings). Random seeds were not fixed.

### Addressing class imbalance

Because recordings with mispronunciation errors were less frequent in the primary dataset, multiple strategies were used to address class imbalance. First, augmentation was used to improve the representation of the minority class. Second, a WeightedRandomSampler was used during training to oversample minority-class recordings when forming minibatches. Third, focal loss (γ = 2.0) was used to emphasize harder examples and reduce the influence of easy majority-class samples.

### Statistical analysis

Descriptive statistics were used to summarize participant characteristics and phoneme-level error prevalence. Group differences by sex, malocclusion status, and age group were initially assessed using χ^2^ tests (or Fisher’s exact test when expected cell counts were <5) for each phoneme. To evaluate whether malocclusion status was associated with a differential phoneme error pattern (i.e., whether the distribution of errors across phonemes differed between malocclusion groups), we also fitted a phoneme-by-group model including a phoneme × malocclusion interaction. Because multiple phoneme-level comparisons were performed, p-values were interpreted with multiplicity control (e.g., Benjamini–Hochberg FDR), with α = 0.05.

Model performance was evaluated using accuracy, precision, recall, and F1-score, along with a confusion matrix. Receiver operating characteristic (ROC) curves were generated, and the area under the curve (AUC) was computed using predicted probabilities for the positive class.

This study is reported in accordance with the Strengthening the Reporting of Observational Studies in Epidemiology (STROBE) guidelines for cross-sectional studies, and the completed checklist is provided as Appendix .

## Results

### Labeling reliability

Inter-rater reliability between the two primary SLP raters showed strong agreement (Cohen’s K = 0.84). Intra-rater reliability, assessed by repeating labeling after a two-week washout period, was also high (SLP1 = 0.92 and SLP2 = 0.89), indicating consistency of labeling over time. Out of the total 1,800 recordings, 125 recordings (6.94%) showed disagreement between the two primary raters. Disagreements between the two primary raters were adjudicated by a third SLP, and the adjudicated labels were used as the final reference standard for training and evaluation.

### Orthodontic findings of the study population

Among the 100 participants, 48 demonstrated normal occlusion. Malocclusion characteristics were overlapping rather than mutually exclusive, with several participants presenting more than one occlusal finding. Crowding (>4 mm) and increased overjet (>4 mm) were the most common findings, observed in 26 and 18 participants, respectively, followed by deep overbite (n = 14), posterior or anterior crossbite (n = 11), and anterior open bite (n = 4).

### Prevalence of speech sound errors

The overall recording-level prevalence of speech sound errors in the study population was 16.15%. When phoneme-category-level mispronunciation prevalence was evaluated, the most frequently affected phoneme category was the grouped dental fricative (“th” category (/θ/ + /ð/), 27.08%), followed by /z/ (23.96%), /f/ (15.58%), /s/ (13.46%), and /ʃ/ (11.59%). The least prevalent mispronunciation was observed for /v/ (8.33%).

### Model training and selection

**Figure 1** shows an overview of the proposed pipeline for automated detection of mispronunciation errors.

**Figure 1:**
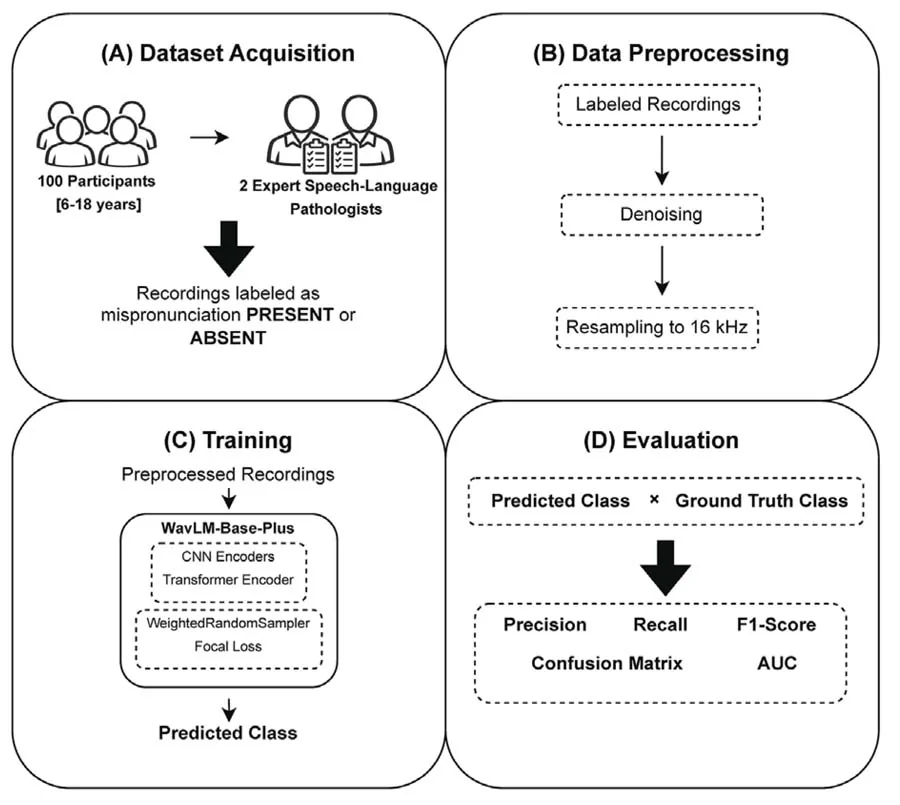
Process of mispronunciation detection. The workflow includes (A) dataset acquisition and expert labeling, (B) audio preprocessing, (C) model training using a WavLM-based architecture, and (D) performance evaluation using standard classification metrics.

The WavLM-based classifier achieved a training accuracy of 0.972 and a validation accuracy of 0.898. Training procedures addressing class imbalance (augmentation, WeightedRandomSampler, and focal loss) were implemented as described in the Methods, and the selected model was evaluated once on the test set. On the held-out test set (n = 253; 163 without mispronunciation error and 90 with mispronunciation error), the model achieved an overall accuracy of 90.9%. The confusion matrix indicated that 160 out of 163 recordings without mispronunciation error were correctly classified (true negatives), with 3 recordings incorrectly classified as mispronunciation error (false positives). Among recordings with mispronunciation error, 70 out of 90 were correctly identified (true positives), while 20 recordings were not detected by the model (false negatives) (**Figure 2**). This pattern reflects a low false-positive rate and a moderate number of false negatives.

**Figure 2:**
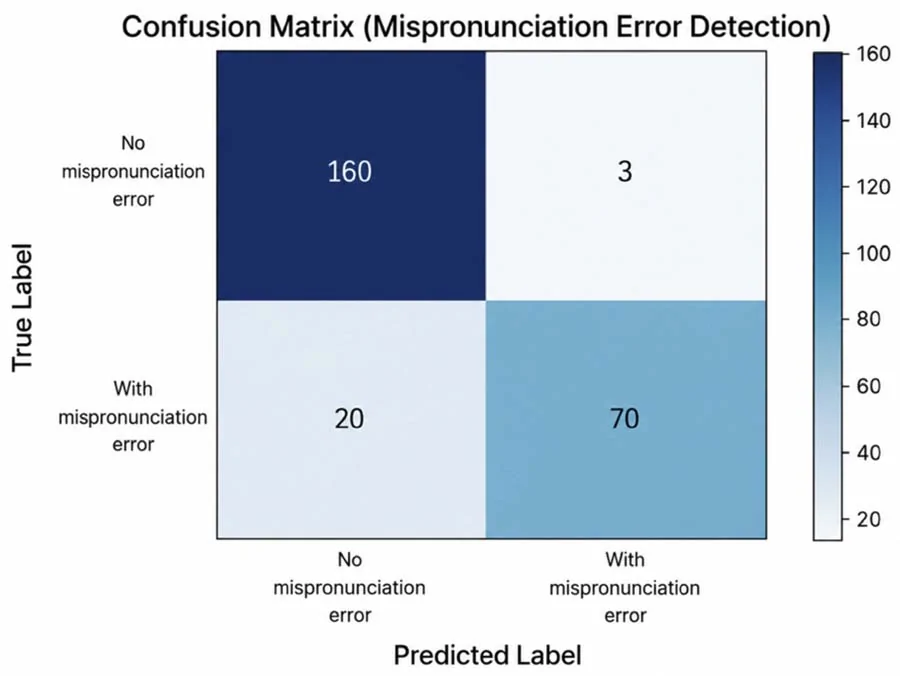
Confusion matrix for the WavLM-based mispronunciation classifier on the test set.

For the detection of mispronunciation error (positive class), sensitivity was 77.8%, and specificity was 98.2%. The F1-score was 0.86 for the mispronunciation error class and 0.93 for the no mispronunciation error class, reflecting high performance in identifying recordings without errors and good performance in detecting mispronunciation errors **(Table 1**).

**Table 1:** Classification performance of the WavLM-based mispronunciation model on the test set (recording-level analysis).

Group	Precision	Recall	F1-score
Without mispronunciation error	0.89	0.98	0.93
With mispronunciation error	0.96	0.78	0.86

The ROC curve summarizes how well the model separates recordings with mispronunciation errors from those without across all possible decision thresholds. In this analysis, the curve remained well above the diagonal “no-discrimination” reference line, indicating that the model consistently achieves a high true-positive rate while maintaining a low false-positive rate across varying classification thresholds (**Figure 3**). The AUC was 0.936, indicating excellent overall discriminative ability; clinically, this suggests the model has a strong capacity to correctly rank recordings from children with mispronunciation errors as higher risk than recordings without errors, regardless of the specific cut-off chosen.

**Figure 3:**
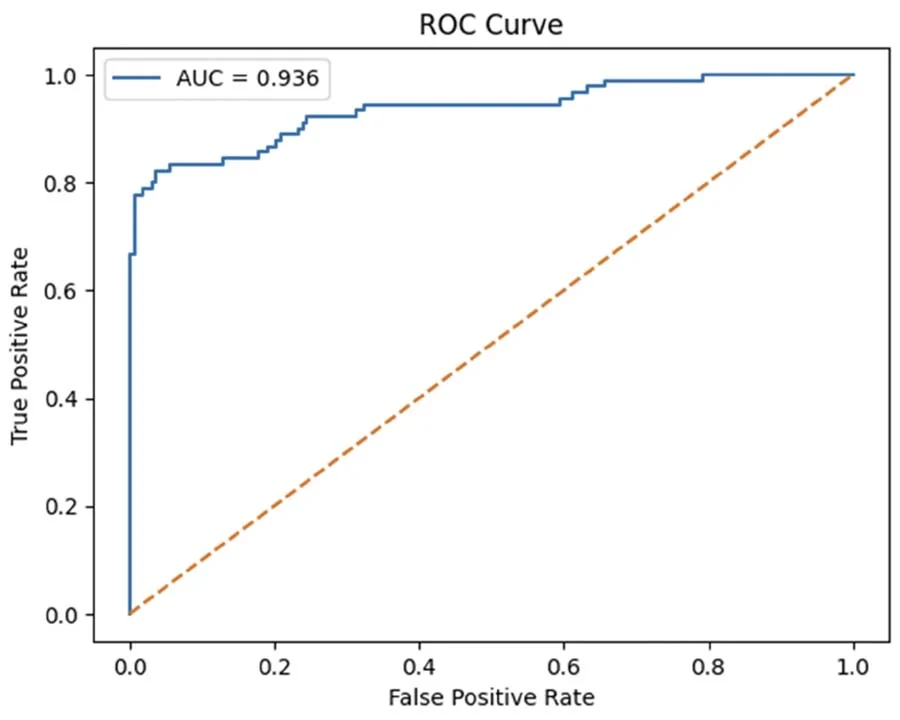
ROC curve for the WavLM-based mispronunciation classifier on the test set.

### Sex comparisons

When stratified by sex, females demonstrated higher prevalence rates for several phonemes; however, inferential testing did not identify statistically significant sex-based differences for any individual phoneme. Chi-square tests showed no significant differences between females and males for all studied phonemes (**Table 2**).

**Table 2:** Gender-based comparison of phoneme-category-level mispronunciation.

Phoneme category	χ^2^ (df = 1)	p-value
/s/	1.29	0.256
“th” category (/θ/ + /ð/)	0.00	1.000
/z/	0.53	0.467
/v/	0.06	0.805
/f/	1.63	0.201
/ʃ/	0.02	0.883

No statistically significant sex-based differences were observed for any phoneme or for total error burden (t(98) = −1.01, p = 0.316).

### Malocclusion comparisons

Phoneme-level mispronunciation prevalence was comparable between participants with and without malocclusion. In phoneme-specific tests, no statistically significant differences were detected for /s/ (χ^2^ = 0.17, p = 0.680), the grouped dental fricative category (“th”; /θ/ + /ð/) (χ^2^ = 0.00, p = 1.000), /z/ (χ^2^ = 0.00, p = 1.000), /v/ (χ^2^ = 0.00, p = 1.000), /f/ (χ^2^ = 1.81, p = 0.179), or /ʃ/ (χ^2^ = 0.06, p = 0.804) (**Table 3**). Consistent with these results, a differential phoneme-pattern analysis (phoneme × malocclusion) did not provide evidence of phoneme-specific shifts in error distribution by malocclusion status in this sample.

**Table 3:** Malocclusion-based comparison of phoneme- category-level mispronunciation errors.

Phoneme category	χ^2^ (df = 1)	p-value
/s/	0.17	0.680
“th” category (/θ/ + /ð/)	0.00	1.000
/z/	0.00	1.000
/v/	0.00	1.000
/f/	1.81	0.179
/ʃ/	0.06	0.804

No statistically significant malocclusion-based differences were observed for any phoneme or for total error burden (t(98) = −0.43, p = 0.669).

### Age comparisons

When stratified by age group (6–9, 10–13, and 14–18 years), no statistically significant differences in mispronunciation prevalence were observed across phonemes. Chi-square tests showed no significant differences among age groups for all studied phonemes (**Table 4**).

**Table 4:** Age group comparison of phoneme- category-level mispronunciation errors.

Phoneme category	χ^2^ (df = 2)	p-value
/s/	1.42	0.492
“th” category (/θ/ + /ð/)	0.88	0.644
/z/	1.15	0.563
/v/	0.67	0.715
/f/	1.73	0.421
/ʃ/	0.94	0.624

No statistically significant age-based differences were observed for any phoneme or for total error burden (t(98) = −0.22, p = 0.715).

## Discussion

In this study, we developed and evaluated a DL-based classifier to automatically detect mispronunciation errors in short, standardized pediatric speech recordings. Using expert SLP adjudication as the reference standard and a transformer-based pretrained speech model fine-tuned for binary classification, the model demonstrated strong overall performance on a held-out test set, with an accuracy of 90.9%, high specificity (98.2%), and good sensitivity (77.8%). The ROC analysis further supported excellent discriminative ability (AUC = 0.936), suggesting that the model can effectively separate recordings with mispronunciation errors from those without across a range of decision thresholds. These findings indicate that modern pretrained speech representations can support automated screening-level detection of mispronunciation errors in children and adolescents, using relatively short audio samples and clinically grounded labels. However, it is important to note that these findings were obtained under controlled conditions, including a standardized word elicitation protocol and recording in a sound-treated environment, which may not fully reflect real-world clinical or community settings.

From a clinical perspective, the observed performance profile is notable for its very high specificity and precision for the positive class. In practical terms, the model rarely misclassified recordings without mispronunciation errors as having mispronunciation errors (false positives were uncommon), which is desirable in screening contexts where unnecessary referrals can burden clinical services and cause avoidable concern for patients and families. This operating profile favors specificity over sensitivity, aligning with a triage-oriented screening role in which false positives may unnecessarily burden referral pathways, whereas missed cases can still be identified through routine clinical observation. At the same time, the sensitivity (77.8%) indicates that some recordings with mispronunciation errors were missed, meaning that a proportion of children with speech errors would not be identified by the model (false negatives). In a clinical screening context, such false negatives may delay referral for comprehensive speech-language evaluation, particularly in cases where subtle articulation errors are not readily detected during routine clinical encounters. This limitation is important when interpreting the model’s role as a screening tool. Therefore, the model is not intended to replace a comprehensive SLP evaluation. Rather, it may be most appropriately positioned as a triage tool that flags likely cases for more detailed assessment, particularly in settings where timely access to specialist evaluation is limited.^31-33^

The AUC of 0.936 suggests that the model has excellent ranking ability, enabling the operating threshold to be tuned depending on the intended clinical use (e.g., shifting toward higher sensitivity when the priority is to minimize missed cases, or toward higher specificity when the priority is to minimize false alarms). This flexibility is aligned with the broader clinical objective of supporting earlier identification of speech-production concerns and referral for comprehensive SLP assessment, including evaluation for SSDs when clinically indicated, given the meaningful educational and psychosocial consequences associated with persistent speech sound difficulties.^1-5^ Beyond threshold adjustment, several strategies may further enhance sensitivity without substantially compromising specificity. These include expanding the diversity and size of the training dataset to better capture subtle and borderline mispronunciation patterns, incorporating phoneme-level or multi-task learning objectives to improve detection of specific error types, and refining data augmentation techniques to better represent acoustic variability. Additionally, future work may explore cost-sensitive learning or adaptive decision thresholds that prioritize detection of clinically relevant errors while maintaining acceptable false-positive rates. Together, these approaches may help optimize the balance between sensitivity and specificity in different clinical screening scenarios.

The strong model performance should also be considered in the context of the study’s labeling approach. Auditory-perceptual assessment remains the primary clinical standard for SSD diagnosis, but it is time-intensive and subject to variability across clinicians and settings.^17-19,34^ Here, we attempted to mitigate this limitation by employing two expert SLPs, adjudicating disagreements through a third SLP, and evaluating both inter- and intra-rater reliability. The resulting reliability estimates were high, supporting the consistency and clinical credibility of the reference standard. This is important because supervised learning is constrained by the quality and stability of the target labels; higher label reliability generally increases the ceiling for achievable model performance and improves interpretability of errors.^35,36^ The relatively small proportion of disagreements requiring adjudication also suggests that the binary task definition (presence vs. absence of mispronunciation error in a recording) was feasible for expert raters under standardized conditions.

AI and DL methods have shown substantial promise in speech-related tasks, including speech recognition and speech error detection.^22-24,37^ However, articulation-focused detection of mispronunciation errors in pediatric populations remains comparatively underrepresented in the applied ML literature, particularly when aligned with clinically interpretable targets and expert-adjudicated labels. Foundational pediatric work has emphasized both the potential and the practical barriers of automatic detection in children; for example, Shahin et al. highlighted key challenges such as developmental variability in child speech, data scarcity, label noise, and the difficulty of generalizing models across ages and elicitation contexts, while also demonstrating early feasibility for disorder detection pipelines.^38^ In parallel, pragmatic “screening-oriented” approaches using engineered acoustic features and conventional ML have been explored to enable clinical decision support in smaller datasets, including feature-engineering frameworks aimed at computer-assisted screening of children with speech disorders.^39^ More recently, there has been a clear shift toward self-supervised and pretrained acoustic representations to reduce reliance on large task-specific pediatric corpora: Ng et al.^40^ showed that SSD detection can be performed using posterior-based speaker representations in child speech, supporting the idea that compact learned representations can capture clinically relevant deviations for classification tasks. Extending this trend, Luan et al. proposed an automatic speech disorder detection (ASDD) system built on self-supervised representations of children’s speech, reinforcing the direction of the field toward scalable and representation-driven pediatric screening systems.^41^ At the evidence-synthesis level, recent mapping work has also consolidated the rapidly expanding landscape of AI tools for speech and language disorders and underscored recurring gaps in clinical validation, interpretability, and real-world deployment pathways.^42^ Against this backdrop, our results add further evidence that transformer-based pretrained acoustic representations can be adapted to clinically meaningful pediatric mispronunciation screening with limited sample sizes through transfer learning, particularly when paired with careful preprocessing (denoising), rigorous expert-adjudicated labels, and explicit imbalance-handling strategies, supporting the broader rationale that AI can augment pattern-recognition workflows in clinical contexts without replacing expert evaluation.

A key methodological element of this work is the use of a pretrained speech model fine-tuned end-to-end for a binary clinical outcome. Pretrained models are increasingly used to improve performance and sample efficiency by leveraging large-scale representations learned from diverse speech corpora.^43^ This is particularly relevant for pediatric datasets, which are often smaller, more heterogeneous, and harder to collect at scale due to stricter privacy standards and limited availability of annotated children’s speech data.^44^

We explored whether mispronunciation error patterns differed by sex, malocclusion status, and age group using phoneme-level chi-square analyses and t-tests comparing mean total error burden. Although the study was not powered for definitive subgroup inference, we did not observe statistically significant differences by sex, age group, or malocclusion status across individual phonemes or total errors. Clinically, these findings are reassuring because they suggest that the model’s performance is less likely to be driven by subgroup-skewed error distributions within this dataset and support the feasibility of applying a single screening model across sexes, age groups, and malocclusion status without immediate need for subgroup-specific decision rules.

Importantly, the present study evaluated mispronunciation using a binary perceptual classification framework and did not differentiate specific error subtypes such as distortions, substitutions, or omissions at the phoneme level. Consequently, the observed phoneme-category error prevalence should not be interpreted as reflecting exclusively malocclusion-related articulatory distortions. Some detected errors may instead represent broader developmental or articulatory variability unrelated to occlusal characteristics within a non-clinical screening population. This may partially explain why fricative categories such as /f/ and /v/ demonstrated observable error prevalence despite the absence of statistically significant phoneme-specific associations with malocclusion status. Future studies incorporating detailed phonetic transcription and subtype-specific error analysis may help better characterize the relationship between articulatory error patterns and dentofacial features.

Importantly, the sample included a broad pediatric-to-adolescent age range (6–18 years). In the present study, age-stratified analyses did not demonstrate statistically significant differences in mispronunciation prevalence across the three developmental groups. This suggests that, within the current dataset and binary classification framework, age-related variability in speech production did not significantly influence the presence/absence labeling used for model training. Given the known developmental variability in speech production across childhood and adolescence, this study was designed as a screening investigation rather than developmental phonetic modeling; therefore, age-related differences in articulation and dentition stage (mixed versus permanent dentition) were not expected to invalidate the binary presence/absence classification of clinically meaningful mispronunciation, particularly under standardized recording procedures and expert SLP adjudication.^45^ This approach is consistent with clinical screening practice, in which expert judgment rather than age-specific phoneme mastery alone determines the need for referral. Although model performance was not evaluated separately by age group in this study due to sample size constraints, future work should include age-stratified performance analysis to further assess model behavior across developmental stages and ensure robustness in diverse pediatric populations.

The relatively higher prevalence of errors observed in the grouped dental fricative category (“th”: /θ/ + /ð/) may reflect the known developmental and articulatory complexity of English dental fricatives, which require precise tongue placement and controlled airflow at the dental interface. Because /θ/ and /ð/ share similar place and manner of articulation despite differing in voicing, they were operationally grouped within a single clinically oriented dental fricative category in the present study. The objective of this grouping was not to distinguish voiced versus voiceless articulatory effects, but rather to evaluate clinically relevant dental fricative production patterns within a standardized screening-oriented framework.

Prior work has shown that while typical speech sound acquisition follows age-dependent trajectories, clinically significant SSDs can be reliably identified across pediatric age ranges using standardized assessments and expert judgment rather than age-specific articulation norms alone.^46^ Nevertheless, larger external datasets will be required to more robustly assess subgroup performance and fairness, including age-stratified evaluation, dialect variation, bilingual or multilingual language exposure, accent-related variability, and broader socio-linguistic factors. In addition, because the elicitation protocol was based on standardized English-language stimuli, external validation across other linguistic and phonological contexts will be necessary before broader cross-language application can be considered. Given the known social and educational impacts of SSDs,^3-5^ demonstrating equitable performance across clinically relevant subgroups is essential for translation into practice.

This study has several strengths that support the credibility and translational relevance of the findings. First, data collection used a standardized, fricative-focused protocol (18 words per participant), resulting in structured, reproducible speech samples across participants, which is advantageous for early-stage model development and interpretation. Second, we used a robust labeling procedure with two expert SLP raters, adjudication by a third SLP in cases of disagreement, and assessment of both inter- and intra-rater reliability, steps that address known limitations of auditory-perceptual evaluation and strengthen the quality of the reference standard.^17-19^ Third, the preprocessing pipeline incorporated denoising, resampling and standardization, and padded batching, reflecting practical considerations for handling real-world recordings while enabling stable minibatch training. Fourth, we addressed class imbalance using complementary strategies (augmentation, weighted sampling, and focal loss), which is commonly recommended in clinical ML settings where minority classes are underrepresented and harder to learn reliably.^21,47^ Finally, model evaluation reported multiple complementary metrics (confusion matrix-derived sensitivity/specificity, precision, F1-score, and AUC), since accuracy alone may be misleading under class imbalance and does not fully capture the clinical trade-offs between missed cases and unnecessary referrals.^48^

Several limitations should be acknowledged. First, the sample size, while sufficient for an initial proof-of-concept study, remains relatively modest for training and evaluating DL models and may limit the robustness of subgroup analyses and generalizability of the findings. In addition, exclusion of participants with previously diagnosed SSDs or prior speech-language therapy likely reduced the prevalence and severity spectrum of mispronunciation errors within the sample; therefore, the present findings primarily reflect detection of milder or previously unidentified mispronunciation patterns within a general pediatric population rather than clinically severe SSD presentations.

Second, recordings were split at the recording level rather than strictly at the participant level. While this choice was aligned with the study objective of detecting mispronunciation errors at the individual word level, it introduces the possibility that recordings from the same participant could appear across training, validation, and test partitions because splitting was performed at the recording level rather than the participant level. This partial speaker overlap could allow the model to learn speaker-specific acoustic characteristics rather than purely phoneme-level features, potentially resulting in overestimation of model performance and reduced generalizability to entirely unseen speakers. Therefore, the reported results should be interpreted with caution in terms of generalizability to unseen speakers. Future studies should prioritize participant-level data splitting and external participant-level validation strategies, in which all recordings from a given participant are confined to a single dataset partition, to ensure full independence between training and evaluation sets and provide a more rigorous assessment of real-world model generalizability.

Additionally, the use of a fixed and structured set of 18 target words, while advantageous for standardization and controlled phoneme elicitation, may limit the variability of speech contexts captured by the model. An additional methodological limitation is that the elicitation protocol included the voiceless postalveolar fricative /ʃ/ but did not include a target word representing the voiced postalveolar fricative /ʒ/. Because /ʃ/ and /ʒ/ share the same place and manner of articulation and differ only in voicing, the absence of /ʒ/ limited the ability of the protocol to evaluate voiced–voiceless contrasts within the postalveolar fricative category. Future studies should include /ʒ/ target words, where developmentally and linguistically appropriate, to provide a more complete assessment of postalveolar fricative production. In naturalistic settings, speech production occurs across more diverse lexical and conversational contexts, which may influence model performance. Finally, all recordings were obtained under standardized conditions in a sound-treated environment, which likely reduced background noise and variability. While this supports internal validity and model training stability, performance in real-world environments with varying acoustic conditions, recording devices, and speech elicitation contexts (e.g., spontaneous or conversational speech) may differ. In clinical and community settings, factors such as background noise, device heterogeneity, and less structured speech input may introduce additional variability that could affect model performance. Future studies should therefore evaluate the model under more ecologically valid conditions, including recordings obtained in routine clinical environments and real-world settings, to assess robustness and support translation into practical screening workflows. Accordingly, the current findings should be interpreted as evidence of performance under controlled experimental conditions rather than direct evidence of real-world clinical implementation readiness.

Despite limitations, the findings support clinically meaningful implications. SSDs are common and can affect academic, psychosocial, and long-term functioning.^1-5^ Because current SSD assessment depends largely on expert auditory-perceptual evaluation, which is time-intensive, can vary across raters, and may be difficult to access promptly in some settings, the present automated classifier may be useful as a triage aid for children with suspected mispronunciation errors or speech-production concerns, prioritizing children for comprehensive SLP evaluation when rapid screening is needed (e.g., school programs, community clinics, or dental/orthodontic settings).^17-20^ Importantly, this tool should be positioned as decision support rather than diagnosis, complementing clinician judgment and improving workflow efficiency rather than replacing expert evaluation, consistent with guidance emphasizing responsible use of AI in health contexts.^49,50^

Future work should focus on 1) external validation across multiple sites, devices, and acoustic environments; 2) prospective evaluation in real-world recording conditions; and 3) richer targets that improve clinical utility (phoneme-level errors, severity, and error subtype classification).^2,17^ Given evidence that malocclusion may influence articulatory placement and the production of fricatives across distinct places of articulation, including labiodental (/f/, /v/), alveolar (/s/, /z/), dental (“th”: /θ/ and /ð/), and postalveolar (/ʃ/) fricatives, the next step is to collect synchronized speech and occlusal data to evaluate whether speech-based models can help inform interdisciplinary referral pathways and earlier intervention in orthodontic practice.^9-16,20^

## Conclusions

This study demonstrates that fine-tuning a pretrained transformer speech model (WavLM) on denoised, standardized English-language pediatric speech recordings can support recording-level detection of mispronunciation errors with strong test performance and excellent discriminative ability under controlled conditions. High expert labeling reliability supports the credibility of the reference standard, and the observed high specificity and precision suggest that the approach may serve as a promising screening-support and triage-oriented decision-support tool to complement, rather than replace, comprehensive SLP assessment. However, additional participant-level and external validation across broader populations, real-world clinical environments, and language-appropriate phonological contexts will be necessary before wider clinical implementation can be considered.
